# Identification of Glutathione S-Transferase (GST) Genes from a Dark Septate Endophytic Fungus (*Exophiala pisciphila*) and Their Expression Patterns under Varied Metals Stress

**DOI:** 10.1371/journal.pone.0123418

**Published:** 2015-04-17

**Authors:** Mi Shen, Da-Ke Zhao, Qin Qiao, Lei Liu, Jun-Ling Wang, Guan-Hua Cao, Tao Li, Zhi-Wei Zhao

**Affiliations:** 1 Key Laboratory of Conservation and Utilization for Bioresources and Key Laboratory of Microbial Diversity in Southwest China, Ministry of Education, Yunnan University, Kunming, Yunnan, China; 2 School of Agriculture, Yunnan University, Kunming, Yunnan, China; 3 School of Life Science, Yunnan University, Kunming, Yunnan, China; CSIR-National Botanical Research Institute, INDIA

## Abstract

Glutathione S-transferases (GSTs) compose a family of multifunctional enzymes that play important roles in the detoxification of xenobiotics and the oxidative stress response. In the present study, twenty four GST genes from the transcriptome of a metal-tolerant dark septate endophyte (DSE), *Exophiala pisciphila*, were identified based on sequence homology, and their responses to various heavy metal exposures were also analyzed. Phylogenetic analysis showed that the 24 GST genes from *E*. *pisciphila* (*EpGST*s) were divided into eight distinct classes, including seven cytosolic classes and one mitochondrial metaxin 1-like class. Moreover, the variable expression patterns of these *EpGST*s were observed under different heavy metal stresses at their effective concentrations for inhibiting growth by 50% (EC_50_). Lead (Pb) exposure caused the up-regulation of all *EpGST*s, while cadmium (Cd), copper (Cu) and zinc (Zn) treatments led to the significant up-regulation of most of the *EpGST*s (*p* < 0.05 to *p* < 0.001). Furthermore, although heavy metal-specific differences in performance were observed under various heavy metals in *Escherichia coli* BL21 (DE3) transformed with *EpGSTN-31*, the over-expression of this gene was able to enhance the heavy metal tolerance of the host cells. These results indicate that *E*. *Pisciphila *harbored a diverse of GST genes and the up-regulated *EpGST*s are closely related to the heavy metal tolerance of *E*. *pisciphila*. The study represents the first investigation of the GST family in *E*. *pisciphila* and provides a primary interpretation of heavy metal detoxification for *E*. *pisciphila*.

## Introduction

Endophytic fungi that cause asymptomatic infections in living plant tissues have been widely studied in various environments [1]. Among the highly diverse group of endophytic fungi, dark septate endophytes (DSE) are characterized by their darkly pigmented and septate hyphae. These melanocratic fungi have been found to ubiquitously colonize the roots of plants growing in extremely heavy metal-contaminated soil [2, 3], and some of them exhibit increased colonization with the increasing levels of heavy metal pollution [4, 5]. It was suggested that these fungi have evolved resistance to heavy metals. One strain of DSE (*Exophiala pisciphila*), which was isolated from an abandoned lead-zinc mine has shown a relatively high tolerance to heavy metals [2]. And this DSE strain could even enhance maize (*Zea mays* L.) tolerance for varied heavy metals when it colonized in the maize root [4]. However, the mechanisms of heavy metal tolerance of DSE are far from elaborated.

One of the ways of metal/metalloid detoxification that occur in almost all living organisms involves metal chelation by small-molecular-mass metabolites, peptides or proteins, such as phytochelatins (PCs) and metallothioneins (MTs) [6–8]. However, the phytochelatin synthetase unigenes which encoded PCs showed no change in expression from the results of our previously analysis based on transcriptome data constructed from *E*. *pisciphila*, furthermore, only three MTs genes were identified from this fungus [9]. Interestingly, it was showed that one of the glutathione S-transferases (GSTs) (EC2.5.1.18) was identified as the most up-regulated unigene in Cd-stressed *E*. *pisciphila* [9].

GSTs are the major detoxifying enzymes that catalyze the nucleophilic conjugation of reduced tripeptide glutathione (GSH; γ-Glu-Cys-Gly) into a wide variety of hydrophobic and electrophilic substrates [10], making them more soluble and easier to excrete [11, 12]. In addition, the activities of some GSTs may overlap with those of the thiol-dependent peroxidases (peroxiredoxins and glutathione peroxidases), which help to reduce damage caused by oxidative stress [13, 14]. GSTs have been found in all prokaryotic and eukaryotic organisms investigated so far, which comprise a complex and widespread enzyme super family. According to their distributions within the cell, three main subfamilies are generally recognized, which include the cytosolic, microsomal (MAPEG) and mitochondrial GST (also known as kappa-class GST) subfamilies [15–17]. It has been subdivided into ever-increasing numbers of classes based on a variety of criteria, including amino acid/nucleotide sequences, and immunological, kinetic, and structural properties. Considering their amino acid sequence identities and several other aspects, nine classes of cytosolic GSTs have been described to date in fungi, including GTT1, GTT2, Ure2p, MAK16, EFB1, etherase-like (recently renamed GSTFuA) [18, 19], phi [20], omega, and glutathionyl hydroquinone reductase (GHR) [12, 21] in addition to a number of uncharacterized classes. However, due to their structural and immunological differences compared with other organisms, a uniform classification of fungal GSTs has not been reached a consensus [12, 17, 20, 21].

Changes in gene expression of GSTs under heavy metal stress have been extensively studied [22–28], while systematic analysis of the whole GSTs repertoire in DSE is still unavailable. Based on the previously established transcriptome data, we conducted a transcriptome-wide annotation, and reported the identifications and classifications of the 24 GST genes from *E*.*pisciphila* (*EpGST*s). We also analyzed the expression patterns of these genes following the exposure of the fungus to various heavy metals. To get a more particular knowledge of the role of GST genes in heavy metal tolerance for *E*. *pisciphila*, *EpGSTN-31*(accession number KJ184545), the most differentially expressed one among the 575 detected genes [9], was selected to analyze its function in heavy metal resistance using transformed *Escherichia coli*. This study provides the first transcriptomic-level insights into the GST gene family of a heavy metal tolerant DSE fungus, and a primary interpretation for its heavy metal tolerant mechanism.

## Materials and Methods

### DSE strain

*Exophiala pisciphila* (H93) was isolated from the roots of *Arundinella bengalensis* which was naturally growing in a waste lead and zinc slag heap in Huize county, Yunnan Province, Southwest China (103°63′ E, 26°55′ N). The sample site was open to everybody without any specific permission from the local government, for there was not any endangered or protected species in this field. This fungus grows well on PDA (potato dextrose agar) medium and has been preserved in the Agricultural Culture Collection Center of China (accession number ACCC32496).

### Identification and phylogenetic analysis of GST genes

Based on the transcritpome database constructed from *E*. *pisciphila* [9], the unique genes encoding putative GSTs of the functional annotations were collected. And then these unigenes were identified using searches against the non-redundant (Nr) protein database of NCBI, and the conserved domains were further confirmed by Pfam (a database of protein families and domains) and Search for Conserved Domains within a protein or coding nucleotide sequence in NCBI. The integrated GST proteins that contained the GST N-terminal domain (PFAM domain PF02798) or GST C-terminal domain (PFAM domain PF00043) were identified. The amino acid sequences of the GSTs were deduced from their cDNA sequences and multiple sequence alignments were performed using the Clustal X (version 1.83) program. The percent amino acid identities of the different GST genes were determined using the DNAStar software (version 5.0). The phylogenetic trees were constructed by the UPGMA method with 1000 bootstrap replications using MEGA 5.0. With the exception of 24 *EpGST*s, the other 77 putative GSTs were extracted from NCBI (S1 Table).

### Expression analysis of GST genes

A quantitative real-time PCR (RT-PCR) analysis of the GST expression patterns in *E*. *pisciphila* was conducted in response to different heavy metals. H93 strains were cultured for 7 d in MMN medium separately supplemented with either one of the four metal ions at the concentrations of their EC_50_ values (111.2 mg L^-1^ Cd^2+^, 1010.0 mg L^-1^ Zn ^2+^, 100.0 mg L^-1^ Cu^2+^, and 800.0 mg L^-1^ Pb^2+^, unpublished data). Total RNA was extracted from the fresh mycelia using the RNAiso Plus Kit (TaKaRa, Japan) according to the manufacturer’s protocol. Then, the quantity and purity of the RNA were determined by UV measurement using the NanoDrop 2000c spectrophotometer (Thermo Scientific, Loughborough, UK). cDNA was synthesized from the total RNA using the PrimeScriptII 1st Strand cDNA Synthesis Kit (TaKaRa, Japan). The primers were designed using Primer 5.0 to amplify the 80bp to 150bp regions of each targeted gene (Table 1). qRT-PCR was performed with the Applied Biosystems 7500 Real-Time PCR System (Applied Biosystems, CA, USA) using SYBR Premix Ex Taq (TaKaRa, Japan) according to the manufacturer’s instructions. The β-tubulin gene was used as an internal control to normalize the quantifications of the *EpGST* expression levels. All of the reaction mixtures were heated at 95°C for 30 s and subjected to 40 PCR cycles at 95°C for 5 s and 60°C for 34 s, and the resulting fluorescence emissions were monitored. PCR reactions without cDNA templates were used as the negative controls. All of the reactions were performed with three biological replicates, and the technical replication of each replicate was conducted independently three times. The relative quantity (RQ) of *Ep*GST expression was analyzed using the 2^-ΔΔCt^ method [29]. Significant differences between the control and treated samples were statistically analyzed using the One-Sample T test in SPSS 12.0 KO (SPSS Inc., Chicago, IL, USA).

**Table 1:** Gene-specific *EpGST*s primers used for qRT-PCR.

GST names	Primers Forward (5' → 3')	Primers Reverse (5' → 3')
*EpUre2p1*	GTATCTACACTGGCGGCGAAC	CCATCCCTTCACCCAGCA
*EpUre2p2*	ACGATCTATCCGAATCCCTCAC	TCAAGTTGCTCCTGTCCTGG
*EpUre2p3*	AGTCCTCCAAGTCTGGGTTCA	GTCGGGCATACATCATTTCC
*EpUre2p4*	AAGAACCCAGAATACCTCGCC	GATTCCCACAGTGTTACATCAGTG
*EpUre2p5*	TCACTCAACATCCACCAACAGA	CTGATGTCAATCTTCTCCACCTTAT
*EpUre2p6*	TATGCCTGGGTTCGTGCTG	CGCCTTCTTTACCGCTGTG
*EpUre2p7*	GAGGACGCACTGAACCGA	CACCCAGGCTTGGAAATG
*EpUre2p8*	ACAAAGAGGCTCTACAAAGTCCTTG	CGTCTACATAGGGTCCGAAAATG
*EpUre2p9*	CTGGATTTCATCGGCACAA	TTTAGGTATTCGGGACTCTTTTGC
*EpGSTN-31*	TCAATCCCAACAGATACCCATG	AATATTTGCATCGTCGTTCCTC
*EpGSTN-32*	GTGAATCGGTCGGCTTCGTA	GCACATCGCCCACCCTC
*EpGSTN-33*	AAGCTCTTCAACGTCACCGATAC	GCGACACGAAGGGTTTGC
*EpGSTN-34*	CGGCGTTCTACCGCTTCA	ATGCCCGAGAAACTCACCC
*EpGSTN-21*	GTCCTGCTCATCAACCCTACC	ATACGCCACATCAAACCTGC
*EpGSTN-22*	ACATCAACAACGGTGTCTACAAGTC	TTCCCGAGGTCTGCTTCTG
*EpGSTN-23*	GGGCTAACGGAAATCATCG	TGGAGATACTCACTGCCGAAC
*EpGSTT1*	TACATGCCAACTATTCACGTCG	ACATTACTCCACCATTTCTGAAGG
*EpGSTT2*	GAAGGATTCACCCTCTACGAGAG	ATTTCTACCGACTGTGCCTGG
*EpGSTT3*	AGTTTCCACCACCCAATCACA	ATCGCAGACACCATCAGCA
*EpGSTG1*	TCCGTCGTCCATAAACAATTG	GGATTCGTCCCAAGGAAGTAA
*EpGSTG2*	CCAACAGGACAGCCCATC	CCCAGTTCTTCGCACAGC
*EpEF1Bγ1*	CCGTAGATGATGCTAAGAGTGCC	GACGAGGAAGGTATGAACAAGGAA
*EpMetaxin11*	CAACAGATAGGCGACCGAAAC	TACCGATGACTGGCATAGCG
*EpGSTZ1*	TCCCTCAGAGTCCGTCCC	CGCCACGCTTTGCCTAA

### Cloning and functional analysis of *EpGSTN-31*

The extraction of total RNA and the synthesis of cDNA were carried out according to the protocols described in the section of Expression analysis of GST genes. Based on the transcriptome sequencing results, a pair of specific primers (forward primer: 5'-GGATCCATGGCGGACGCACGGCC-3' (*BamHI*), reverse primer: 5'-GTCGACCTATAGCATTGACTTCGCAGTAGGCC-3' (*SalI*) (underlined)) was designed to amplify the cDNA sequence that encoded the mature *EpGSTN-31*. The PCR products were purified, and the ORF (open reading frame) of *EpGSTN-31* was ligated into the pMD18-T simple cloning vector and sequenced. Then, the recombinant pMD18-T simple vector was digested with the restriction enzymes *BamHI* and *SalI*. The *EpGSTN-31* ORF fragment was subsequently subcloned into the expression vector pGEX-4T-1 with *BamHI* and *EcoRI* recognition sites using T4 DNA Ligase (Fermentas, Hanover, MD, USA) according to the manufacturer’s protocol. The recombinant vector with the correct orientations of the insert pGEX-4T-1-EpGSTN-31 was transformed into *E*. *coli* BL21 (DE3) cells. The cells transfected with pGEX-4T-1 were used as negative controls. Both transfected BL21 strains were separately inoculated into 100 ml LB liquid medium and induced with 200 mg L^-1^ isopropyl β-D-thiogalactopyranoside (IPTG). For the growth curve assay of the cells under heavy metal stresses, the BL21 strains were cultured for 24 h in the above medium separately supplemented with either of the four metal ions at different concentrations (50 mg L^-1^ Cd^2+^, 100 mg L^-1^ Zn ^2+^, 70 mg L^-1^ Cu^2+^, and 100 mg L^-1^ Pb^2+^). Growth was measured and analyzed by monitoring the increase in absorbance at 600 nm at 4 h intervals for 24 h. Each treatment was conducted independently four times. Significant differences between the control cells and the cells containing the recombinant plasmid subjected to the different heavy metal stresses were assessed using the Independent-Samples T test with SPSS 12.0 KO (SPSS Inc., Chicago, IL, USA).

## Results

### Identification and classification of *E*. *pisciphila* GST genes

**Fig 1:**
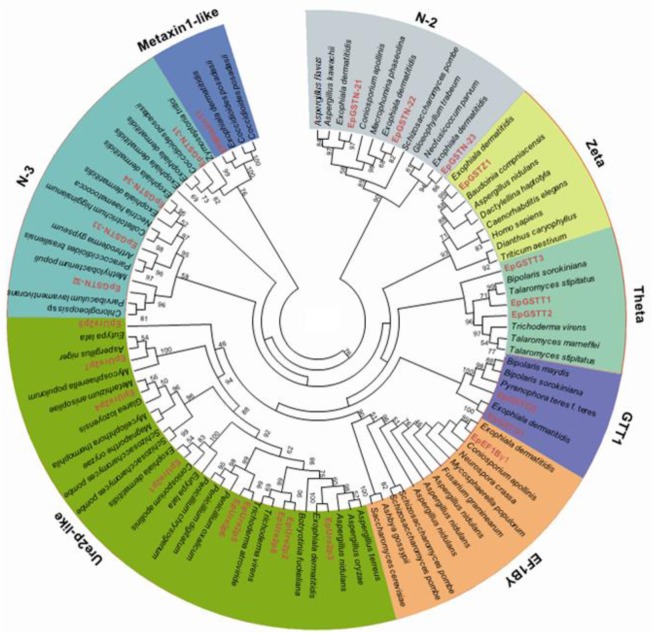
Phylogenetic relationships among the 24 *EpGST*s. Seventy fungal proteins and seven additional full-length proteins come from plants, animals, bacteria and nematodes. The accession numbers of all these genes are listed in S1 Table. The tree begins with the GST of the N-2 class from *Aspergillus flavu* and extends clockwise. The amino acid sequences were aligned using Clustal W, and a UPGMA tree was generated using the MEGA 5.0 software with 1000 bootstrap replicates. The numbers at each node of the phylogenetic tree represent the bootstrap values, and only those bootstrap values greater than 45% are shown. The red letters represent the GSTs from *E*. *pisciphila*. The eight clades harboring the *EpGST*s are shaded in different colors.

A total of 24 non-redundant gene loci were predicted to code for putative full-length GST proteins in *E*. *pisciphila*. The ORF, protein lengths and modular domain structure of all the 24 GST genes are provided in S1 Fig and S2 Table. The proteins included in the Ure2p-like, N-2 (GST_N family, unknown subfamily 2), N-3 (GST_N family, unknown subfamily 3), zeta, theta, GTT1, EF1Bγ, and metaxin 1-like classes were designated as *EpUre2p*, *EpGSTN-2*, *EpGSTN-3*, *EpGSTZ*, *EpGSTT*, *EpGSTG*, *EpEF1Bγ* and *EpMetaxin1*, respectively (Fig 1). Twenty three full-length genes encoding putative cytosolic GSTs and one encoding a mitochondrial GST (*EpMetaxin11*) were identified. These newly identified members were named according to their sequence identities, and all of the *EpGST* sequences have been deposited into GenBank and assigned accession numbers (S2 Table).

The evolutionary relationship among the *E*. *pisciphila* GST proteins was demonstrated in the phylogenetic tree generated using the MEGA 5.0 software with their full-length protein sequences (Fig 1). It was showed that the *EpGST* family could be divided into eight classes with high bootstrap support. Based on the phylogenetic tree analysis, the *EpUre2p* had the largest number of GST genes (nine members), followed by the *EpGSTN-3* (four members), *EpGSTN-2* (three members), *EpGSTT* (three members), *EpGSTG* (two members), *EpGSTZ* (one member), *EpEF1Bγ* (one member) and *EpMetaxin1* (one member).

The different GST transcripts of *E*. *pisciphila* were aligned to one another for the assessments of their amino acid identities. The pairwise comparisons of the GST protein sequences revealed considerable significant differences, with overall identities ranging from 1.5% to 60.6% (S3 Table). Relatively higher identities were found among members of the same class; for example, *EpUre2p* generally showed the highest pairwise sequence identity, which varied from 16.0% to 60.6%. However, for the other classes, most of identities were less than 20.0% (S3 Table).

### Expression patterns of *EpGST*s under heavy metal stresses

**Fig 2:**
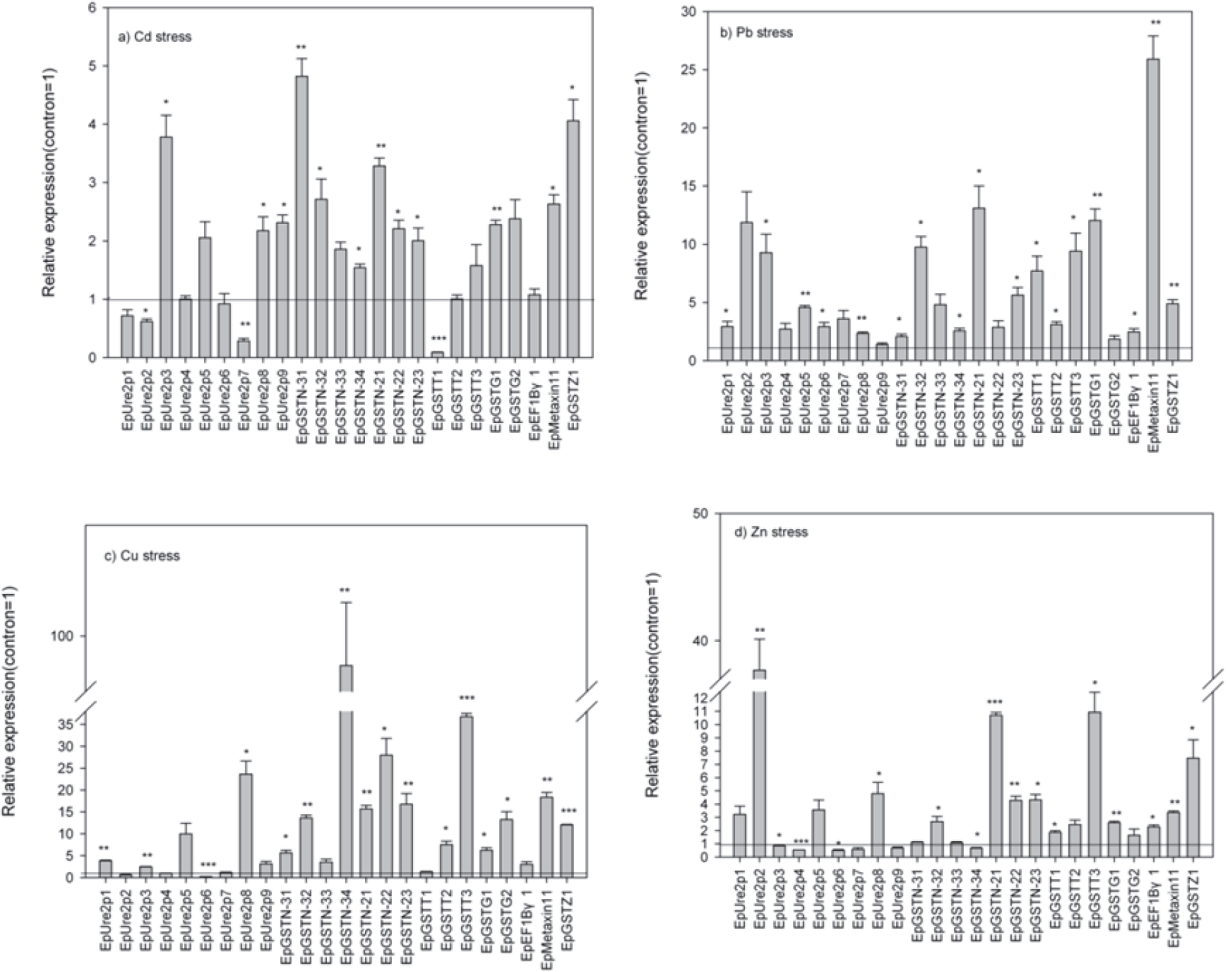
qRT-PCR of *EpGST*s expression under stress of various heavy metals (Cd, Pb, Cu, and Zn). *Ep*GSTs mRNA expression levels were calculated relative to β-tublin expression and are shown as the mean ± SE (n = 3). Asterisks indicate significant differences (*p* < 0.05*, *p* < 0.01**, *p* < 0.001***) compared to the control according to One-Sample T test.

According to the qRT-PCR results, the GST mRNAs in *E*. *pisciphila* showed differential expression patterns during Cd exposure at its EC_50_ concentration (Fig 2A; S4 Table). The expression levels of all genes in the classes of *EpGSTN-3*, *EpGSTN-2*, *EpGSTG*, *EpMetaxin1*, and *EpGSTZ* were significantly up-regulated (*p* < 0.05 to *p* < 0.01, Fig 2A; S4 Table), except for those of *EpGSTN-33* and *EpGSTG2* genes which showed no significant change. And the highest expression was observed in *EpGSTN-31* with the relative expression of 4.82-fold of its control (*p* < 0.01, Fig 2A; S4 Table). However, as concerned to the class of *EpUre2p*, the expression varied greatly among the genes. *EpUre2p3*, *EpUre2p8*, and *EpUre2p9* transcripts were significantly up-regulated (*p* < 0.05 to *p* < 0.01), while the expression levels of *EpUre2p2*, and *EpUre2p7* were significantly down-regulated (*p* < 0.05 to *p* < 0.01), and no significant change was observed in the expression of other genes in this class (Fig 2A; S4 Table).

When the fungal strain were exposed to Pb at its EC_50_ concentration, none of the expression were significantly down-regulated of the 24 *EpGST*s genes (*p* < 0.05 to *p* < 0.001, Fig 2B; S4 Table). Furthermore, about 17 *EpGST*s were significantly up-regulated, and the *EpMetaxin11* showed the highest increase with a 25.9- fold compared to the control (*p* < 0.05 to *p* < 0.001, Fig 2B; S4 Table).

On the exposure of Cu at its EC_50_ concentration, the expression patterns of *EpGST*s showed somewhat similar with the exposure to Cd. The expression levels of all genes in the classes of *EpGSTN-3*, *EpGSTN-2*, *EpGSTT*, *EpGSTG*, *EpMetaxin1*, and *EpGSTZ* were significantly up-regulated (*p* < 0.05 to *p* < 0.001, Fig 2C; S4 Table), except for those of *EpGSTN-33* and *EpGSTT1* genes which showed no significant change. And the maximum increase expression (97.3 fold) was observed in *EpGSTN-34* (*p* < 0.01, Fig 2C; S4 Table). Similarly, the genes in *EpUre2p* showed complex responses to Cu stress. *EpUre2p1*, *EpUre2p3*, and *EpUre2p8* transcripts were significantly up-regulated (*p* < 0.05 to *p* < 0.01), while the expression level of *EpUre2p6* was significantly down-regulated (*p* < 0.001), and no significant change was observed in the expression of other genes in this class (Fig 2C; S4 Table).

The GST mRNAs in Zn-exposed *E*. *pisciphila* showed different expression profiles in this study. For example, the expression levels of 12 *EpGST*s were significantly up-regualted, and the highest expression (37.7 fold) was observed in *EpUre2p2* (*p* < 0.05 to *p* < 0.001, Fig 2D; S4 Table). As concerned to *EpUre2p3*, *EpUre2p4*, *EpUre2p6*, and *EpGSTN-34*, their expression levels dropped obviously, and *EpUre2p6* showed the lowest expression with a 0.51- fold compared to the control (*p* < 0.05 to *p* < 0.001, Fig 2D; S4 Table).

### Heavy metal tolerance analysis of transformed *E*. *coli*

**Fig 3:**
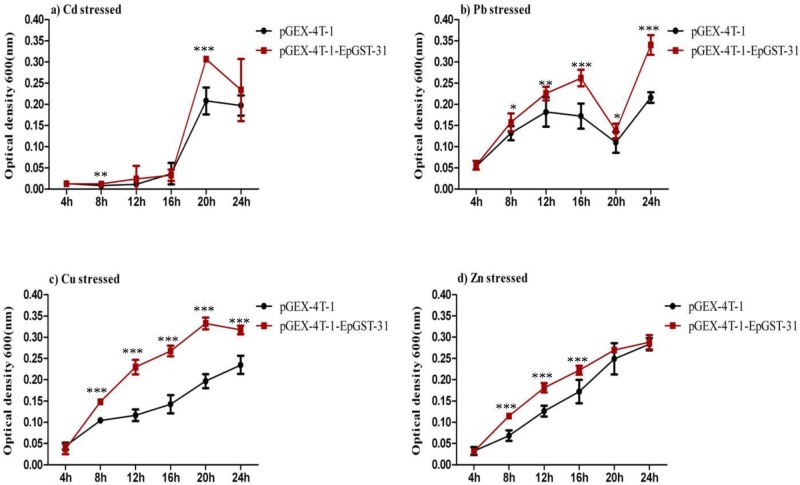
Growth curves of the transformed BL21 strains with empty pGEX-4T-1(negative control) and recombinant pGEX-4T-1-EpGSTN-31 plasmids. A total of 200 mg L^-1^ IPTG was added to each culture to induce the expression of the recombinant protein. (a) 50 mg L^-1^ Cd^2+^ (b) 100 mg L^-1^ Pb^2+^ (c) 70 mg L^-1^ Cu^2+^ and (d) 100mg L^-1^ Zn ^2+^. Each treatment was conducted independently four times. Asterisks indicate significant differences (*p* < 0.05*, *p* < 0.01**, *p* < 0.001***) compared to the control according to Independent-Samples T test.

There are currently no reports in the literature describing heavy metal tolerance in association with the GST N-3 class. According to the results of transcriptome analysis, the top differentially expressed gene was *EpGSTN-31* among 575 detected genes [9]. In addition, the qRT-PCR analysis revealed that *EpGSTN-31* was the most highly expressed among the 24 *EpGST*s under Cd stress. Taken together, we carried out the functional verification of the *EpGSTN-31*. The results (Fig 3) indicated that compared with the control strain containing the empty pGEX-4T-1 vetors, the cell growth of the transformed BL21 strain expressing *EpGSTN-31* was significantly enhanced at most of the exposure times under various heavy metal stresses. However, the transformed strain responded differently to Pb, Zn, Cu and Cd treatments; *EpGSTN-31* appeared to confer better Pb and Cu tolerances compared to those of Cd and Zn, particularly following long-term exposure (Fig 3). In addition, we observed maximal levels of heavy metal tolerance in the transformed cells after 8 h of exposure to Cu stress compared to the control cells, but the transformed cells under Cd and Pb stresses did not show maximal tolerance levels until the 20th and 24th hours, respectively. Taken together, these results demonstrated that the over-expression of *EpGSTN-31* was able to enhance the heavy metal tolerance of the host cells, but heavy metal-specific differences in performance were observed among the various heavy metals.

## Discussion

The *EpGST*s were divided into the *EpUre2p*, *EpGSTN-2*, *EpGSTN-3*, *EpGSTZ*, *EpGSTT*, *EpGSTG*, *EpEF1Bγ* and *EpMetaxin1* according to the phylogenetic tree constructed using 101 full-length GST proteins. As the relationship between fungal GSTs and the mainstream classification system remains unclear, some of the *EpGSTs* do not fit easily into any of the previously characterized classes of fungi based on immunological, sequence or catalytic criteria [21], such as those of the theta and metaxin 1-like classes. These difficulties in classification suggested the divergences and complex evolutionary relationships of the fungal GSTs compared to those of other organisms [12, 30]. In addition, discrepancies in the GST classification standards also affected its classifications. Traditionally, two proteins belong to the same class if they share more than 40% identities and if they share less than 20% identities with isoenzymes belonging to different classes [16]. Nevertheless, based on these primary sequence criteria only, many non-canonical GST groups have emerged, particularly in fungi, increasing the complexities of GST classifications. For example, a few protein families are typically classified as GSTs (EF1Bγ, MAK16), based on structural similarities but, not on the existence of glutathione-dependent activities [12].

Among the 8 classes of *EpGST*s, the members of the N-3 class surprisingly did not assemble into a natural branch in the phylogenetic tree. According to the GSTs database from NCBI, N-3 is an unknown class of the GST family and composed of uncharacterized bacterial proteins. Thus, detailed information of these proteins is extremely limited and it is unclear whether the N-3 class is a natural taxonomic unit till now. The phylogenetic tree revealed that the N-3 members were not closely related to one another, and these findings were supported by their sequence identities. As S5 Table showed, the pairwise sequence identities of the 17 members in the N-3 class ranged from 1.8% to 74.3%, thus this class was considerably diverse. Amino acid sequence variations in the conserved domain for the N-3 class may lead to its separation in the constructed phylogenetic tree. Thus, further investigations of this class should be performed to delineate its divergence. In addition, the Metaxin1-like class clustered with 4 members of the N-3 class with high bootstraps support, indicating the close genetic relationship of these two classes, at least in association with the branch containing *EpGSTN-31*.

The pairwise comparisons of the 24 *EpGST*s revealed that the GSTs in *E*. *pisciphila* showed extremely low pairwise sequence diversities with the exception of those in the *EpUre2p* (S3 Table). Furthermore, the percent identities of the 13 GSTs from *Exophiala dermatitidis* were also considerably low, ranging from 3.3% to 55.6% (S6 Table). However, a pairwise comparison of rice (*Oryza sativa*) GST protein sequences has described overall percent identities ranging from 8% to 92% [31], and eight hemerythrin GSTs from *Physcomitrella patens* have been reported with 38.0% to 94.7% pairwise sequence identities [10], which are more conserved compared with the *EpUre2p*. Taken together, fungal GSTs, at least in *E*. *pisciphila and E*. *dermatitidis*, possess greatly divergent amino acid sequences.

A dominant species-specific GST class has been detected in previous reports [32, 33]. In the present study, the Ure2p-like class was the largest class in *E*. *pisciphila* with nine members. The number of isoforms in each class differed considerably on a species-specific basis, e.g., the Ure2p-like class was over-represented in *Phanerochaete chrysosporium* (nine sequences), *Postia placenta* (sixteen sequences) and *Phanerochaete carnosa* (21 sequences), respectively, whereas only one Ure2p-like sequence was detected in *Laccaria bicolor* and *Cryptococcus neoformans* [12]. A previous bioinformatic analysis suggested that the prion-like and GST-like domains of Ure2p diverged separately [34], indicating the diverse functions of this class. These previous reports of Ure2p class were in accordance with our findings of the variable responses of this class to the heavy metals compared to those of the other classes in *E*. *pisciphila*, particularly following exposures to Cd, Cu and Zn stresses. Morel et al. [20] also suggested that the expansion and diversification of Ure2p in *Agaricomycotina* was associated with differing environmental conditions. For example, the Ure2p9 and Ure2p6 genes in *Phanerochaete chrysosporium* were induced in the presences of aspen and pine, respectively [35], while Ure2p4 and Ure2p6 showed specific expression after polycyclic aromatic compound treatments [36]. Though the heavy metal treatments led to the diverse responses of *EpUre2p* genes, the relative expression of *EpUre2p8* was significantly up-regulated under either one of the four heavy metal stresses (Fig 2). It was suggested that this gene is closely related to the heavy metal tolerance of *E*. *pisciphila*. Fungal Ure2p proteins did not show detectable GST activities using the standard substrate 1-chloro-2, 4-dinitrobenzene (CDNB); however, they did exhibit GSH-dependent peroxidase activities [37]. It has been reported that fungal Ure2p members participated in heavy metal ion and oxidant detoxification [38].

The zeta and theta classes have very specific activities toward xenobiotics [10]. The zeta class has been shown to differ from the other GST classes due to its lack of GSH conjugation activity and GSH peroxidase activity; members of this class are involved in GSH-dependent tyrosine catabolism [39]. In this study, we observed that *EpGSTZ1* was significantly up-regulated following the treatments with the four heavy metals, indicating that it played a role in some unknown mechanism to combat heavy metal stress. Members of the theta class had limited transferase activities towards xenobiotics but were highly active GSH-dependent peroxidases that were involved in oxidative stress metabolism [40]. Therefore, the GSH-dependent peroxidase activities of *EpGSTT1*, *EpGSTT2* and *EpGSTT3*, which were significantly up-regulated, were beneficial for reducing ROS damage and contributed to the heavy metal tolerance of *E*. *pisciphila*. Similar to that observed within the theta class of *E*. *pisciphila*, *EpMatxin11* was significantly up-regulated following the different heavy metal treatments. Previous research has indicated that this GST was not enzymatically active [41]. Therefore, *EpMatxin11* may reduce the negative effects of heavy metal stress by unknown means. There are two GTT1 members in *E*. *pisciphila* that are able to act on standard GST substrates, such as CDNB, and provide protection against oxidants and oxidative stress through their glutathione peroxidase activities [42–44]. Additionally, *EpEF1Bγ1* has been detected in fungi; however, it is unclear whether the EF1Bγ protein has retained any GST-type catalytic activities, although this function has been predicted by motif database searches [45, 46].

Interestingly, we observed that the Cd, Cu and Zn exposures caused both the up- and down-regulations of the *EpGST*s, while all of the *EpGST*s were up-regulated under the Pb stress. Previous research has shown that *E*. *pisciphila* was able to accumulate 20.00% Pb, 15.56% Zn and 3.57% Cd of its biomass indicating that this fungus was more tolerant to Pb [2]. These results were in accordance with our findings of the up-regulations of all of the *EpGST*s under Pb stress, and the up-regulations of only portions of the *EpGSTs* following the Cd, Cu and Zn treatments. Coincidently, we also observed that the *EpGSTN-31*-transformed *E*. *coli* (BL21) showed significant Pb tolerance compared with the control and also compared with the tolerances to Zn, Cu and Cd at 24 h (the end of exposure). In general, GST expression is considered to be closely related to the type and concentration of the heavy metal and the duration of the treatment time of heavy metal [13, 47, 48]. While, only the type of heavy metal was taken into account in this study. Therefore, additional factors should be examined in the near future to allow for the comprehensive understanding of the expression characteristics of the *EpGST*s.

In conclusion, twenty four GST genes from the transcriptome of *E*. *pisciphila* were identified based on sequence homology in this study. They were divided into eight classes, among which the *EpUre2p* were the most highly represented, with nine members. In contrast with Cd, Cu and Zn stresses, Pb stress led to the up-regulation of all *EpGST*s. However, Cd, Cu and Zn exposures led to the differential expressions of some members of the *EpUre2p*. In addition, *EpGSTN-31* was shown to be able to enhance heavy metal tolerance in *E*. *coli* BL21 cells. Thus, by identifying the *EpGST*s and examining their expressions under varied heavy metal stresses and the heavy metal tolerance of one GST, it has been revealed that most *EpGST*s are closely related to the heavy metal tolerance of *E*. *pisciphila*.

## Supporting Information

S1 Fig The modular domain structure and protein lengths of all the 24 *EpGST*s. (TIF)

S1 Table The class, accession number and source organnisms of the 101 GSTs that was used to construct the phylogenetic tree, including 24 *Exophiala pisciphila* GSTs and 70 fungal proteins and other 7 full-length proteins from plants, animals, bacteria and nematode. (DOC)

S2 Table Nomenclature and sequence information of the 24 *EpGST*s. (DOC)

S3 Table Percentage of identities of amino acid residues within the GSTs of *E*. *pisciphila*. (DOC)

S4 Table Average values of relative expression of the 24 *EpGST*s. (DOC)

S5 Table Percentage of identities of amino acid residues within the 17 members in N-3 class. (DOC)

S6 Table Percentage of identities of amino acid residues within the 13 GSTs in *Exophiala dermatitidis*. (DOC)
