# Supplementary figures and images for: Identification of Glutathione S-Transferase (GST) Genes from a Dark Septate Endophytic Fungus (Exophiala pisciphila) and Their Expression Patterns under Varied Metals Stress
Source: PLoS One. 2015 Apr 17;10(4):e0123418. doi: 10.1371/journal.pone.0123418 (PMC4401685; doi:10.1371/journal.pone.0123418)

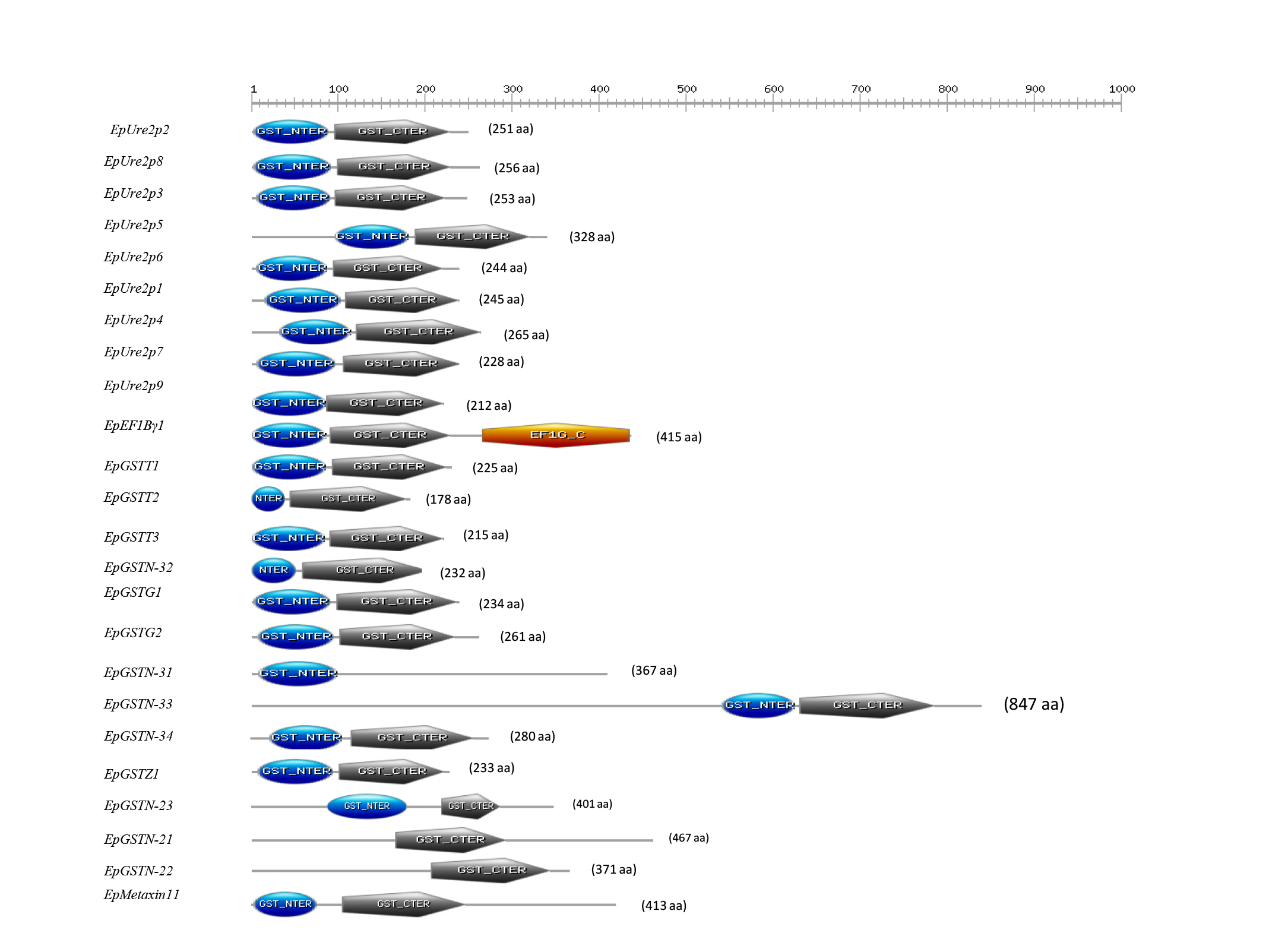

Supplement: S1 Fig — (TIF) [file pone.0123418.s001.tif]
