# Supplementary material for: Identification of Glutathione S-Transferase (GST) Genes from a Dark Septate Endophytic Fungus (Exophiala pisciphila) and Their Expression Patterns under Varied Metals Stress
Source: PLoS One. 2015 Apr 17;10(4):e0123418. doi: 10.1371/journal.pone.0123418 (PMC4401685; doi:10.1371/journal.pone.0123418)
Supplement: S1 Table — (DOC) [file pone.0123418.s002.doc]

**S1 Table. The class, accession number and source organnisms of the 101 GSTs that was used to construct the phylogenetic tree, including 24 *Exophiala pisciphila* GSTs and 70 fungal proteins and other 7 full-length proteins from plants, animals, bacteria and nematode**

| GST class | Accession number | Source organisms |
| --- | --- | --- |
| N-2 | XP_002378175.1 | *Aspergillus flavus* |
|  | GAA86356.1 | *Aspergillus kawachii* |
|  | EHY59473.1 | *Exophiala dermatitidis* |
|  | KJ862285 | *Exophiala pisciphila* |
|  | EON60824.1 | *Coniosporium apollinis* |
|  | EKG13394.1 | *Macrophomina phaseolina* |
|  | EHY52776.1 | *Exophiala dermatitidis* |
|  | KJ862286 | *Exophiala pisciphila* |
|  | NP_588171.1 | *Schizosaccharomyces pombe* |
|  | EPQ56880.1 | *Gloeophyllum trabeum* |
|  | EOD50997.1 | *Neofusicoccum parvum* |
|  | EHY51929.1 | *Exophiala dermatitidis* |
|  | KJ862287 | *Exophiala pisciphila* |
| Zeta | EHY55296.1 | *Exophiala dermatitidis* |
|  | KJ862295 | *Exophiala pisciphila* |
|  | EMD00515.1 | *Baudoinia compniacensis* |
|  | XP_659499.1 | *Aspergillus nidulans* |
|  | EPS43246.1 | *Dactylellina haptotyla* |
|  | NP_509962.1 | *Caenorhabditis elegans* |
|  | AAB96392.1 | *Homo sapiens* |
|  | P28342.1 | *Dianthus caryophyllus* |
|  | O04437.1 | *Triticum aestivum* |
| Theta | KJ862290 | *Exophiala pisciphila* |
|  | EMD68562.1 | *Bipolaris sorokiniana* |
|  | XP_002340542.1 | *Talaromyces stipitatus* |
|  | KJ862288 | *Exophiala pisciphila* |
|  | KJ862289 | *Exophiala pisciphila* |
|  | EHK23094.1 | *Trichoderma virens* |
|  | XP_002145828.1 | *Talaromyces marneffei* |
|  | XP_002482063.1 | *Talaromyces stipitatus* |
| GTT1 | EMD86126.1 | *Bipolaris maydis* |
|  | EMD67176.1 | *Bipolaris sorokiniana* |
|  | XP_003307049.1 | *Pyrenophora teres f. teres* |
|  | KJ862292 | *Exophiala pisciphila* |
|  | EHY59024.1 | *Exophiala dermatitidis* |
|  | KJ862291 | *Exophiala pisciphila* |
| EF1Bγ | EHY56652.1 | *Exophiala dermatitidis* |
|  | KJ862293 | *Exophiala pisciphila* |
|  | EON62153.1 | *Coniosporium apollinis* |
|  | XP_961215.1 | *Neurospora crassa* |
|  | EMF15588.1 | *Mycosphaerella populorum* |
|  | XP_387577.1 | *Fusarium graminearum* |
|  | XP_664167.1 | *Aspergillus nidulans* |
|  | XP_681076.1 | *Aspergillus nidulans* |
|  | XP_659199.1 | *Aspergillus nidulans* |
|  | NP_587885.1 | *Schizosaccharomyces pombe* |
|  | P40921.1 | *Schizosaccharomyces pombe* |
|  | NP_984243.2 | *Ashbya gossypii* |
|  | NP_012842.1 | *Saccharomyces cerevisiae* |
| Ure2p-like | XP_001819791.1 | *Aspergillus oryzae* |
|  | XP_001213789.1 | *Aspergillus terreus* |
|  | CBF76508.1 | *Aspergillus nidulans* |
|  | KJ862275 | *Exophiala pisciphila* |
|  | EHY60945.1 | *Exophiala dermatitidis* |
|  | AAG43132.1 | *Botryotinia fuckeliana* |
|  | KJ862274 | *Exophiala pisciphila* |
|  | KJ862280 | *Exophiala pisciphila* |
|  | EHK23903.1 | *Trichoderma virens* |
|  | EHK44330.1 | *Trichoderma atroviride* |
|  | KJ862277 | *Exophiala pisciphila* |
|  | KJ862278 | *Exophiala pisciphila* |
|  | EPS29865.1 | *Penicillium oxalicum* |
|  | EKV07338.1 | *Penicillium digitatum* |
|  | XP_002561977.1 | *Penicillium chrysogenum* |
|  | EMR64607.1 | *Eutypa lata* |
|  | EON67697.1 | *Coniosporium apollinis* |
|  | KJ862273 | *Exophiala pisciphila* |
|  | EHY56510.1 | *Exophiala dermatitidis* |
|  | NP_588298.1 | *Schizosaccharomyces pombe* |
|  | NP_588517.1 | *Schizosaccharomyces pombe* |
|  | EAQ71536.1 | *Magnaporthe oryzae* |
|  | XP_003659301.1 | *Myceliophthora thermophila* |
|  | EHL02127.1 | *Glarea lozoyensis* |
|  | KJ862276 | *Exophiala pisciphila* |
|  | EFY97782.1 | *Metarhizium anisopliae* |
|  | EMF09857.1 | *Mycosphaerella populorum* |
|  | KJ862279 | *Exophiala pisciphila* |
|  | CAK39793.1 | *Aspergillus niger* |
|  | EMR67874.1 | *Eutypa lata* |
|  | KJ862281 | *Exophiala pisciphila* |
| N-3 | WP_016877175.1 | *Chlorogloeopsis* sp |
|  | YP_001412927.1 | *Parvibaculum lavamentivorans* |
|  | KJ862282 | *Exophiala pisciphila* |
|  | YP_001925563.1 | *Methylobacterium populi* |
|  | EEH45625.1 | *Paracoccidioides brasiliensis* |
|  | XP_003170769.1 | *Arthroderma gypseum* |
|  | KJ862283 | *Exophiala pisciphila* |
|  | CCF35233.1 | *Colletotrichum higginsianum* |
|  | XP_003045069.1 | *Nectria haematococca* |
|  | EHY54854.1 | *Exophiala dermatitidis* |
|  | KJ862284 | *Exophiala pisciphila* |
|  | EHY60173.1 | *Exophiala dermatitidis* |
|  | EHY56712.1 | *Exophiala dermatitidis* |
|  | EHY57213.1 | *Exophiala dermatitidis* |
|  | EON62413.1 | *Coniosporium pollinis* |
|  | KJ184545 | *Exophiala pisciphila* |
|  | XP_003853521.1 | *Zymoseptoria tritici* |
| Metaxin1-like | KJ862294 | *Exophiala pisciphila* |
|  | EHY52537.1 | *Exophiala dermatitidis* |
|  | EFW15805.1 | *Coccidioides posadasii* |
|  | XP_003065220.1 | *Coccidioides posadasii* |
