# Supplementary material for: Identification of Glutathione S-Transferase (GST) Genes from a Dark Septate Endophytic Fungus (Exophiala pisciphila) and Their Expression Patterns under Varied Metals Stress
Source: PLoS One. 2015 Apr 17;10(4):e0123418. doi: 10.1371/journal.pone.0123418 (PMC4401685; doi:10.1371/journal.pone.0123418)
Supplement: S2 Table — (DOC) [file pone.0123418.s003.doc]

**S2 Table. Nomenclature and sequence information of the 24 *EpGST***s

| GST class | GST name | Open reading frame length | Protein length | Accession number |
| --- | --- | --- | --- | --- |
| Ure2p-like | *EpUre2p1* | 738 | 245 | KJ862273 |
|  | *EpUre2p2* | 756 | 251 | KJ862274 |
|  | *EpUre2p3* | 762 | 253 | KJ862275 |
|  | *EpUre2p4* | 798 | 265 | KJ862276 |
|  | *EpUre2p5* | 987 | 328 | KJ862277 |
|  | *EpUre2p6* | 735 | 244 | KJ862278 |
|  | *EpUre2p7* | 687 | 228 | KJ862279 |
|  | *EpUre2p8* | 771 | 256 | KJ862280 |
|  | *EpUre2p9* | 639 | 212 | KJ862281 |
| N-3 | *EpGSTN-31* | 1104 | 367 | KJ184545 |
|  | *EpGSTN-32* | 699 | 232 | KJ862282 |
|  | *EpGSTN-33* | 2544 | 847 | KJ862283 |
|  | *EpGSTN-34* | 843 | 280 | KJ862284 |
| N-2 | *EpGSTN-21* | 1404 | 467 | KJ862285 |
|  | *EpGSTN-22* | 1116 | 371 | KJ862286 |
|  | *EpGSTN-23* | 1206 | 401 | KJ862287 |
| Theta | *EpGSTT1* | 678 | 225 | KJ862288 |
|  | *EpGSTT2* | 537 | 178 | KJ862289 |
|  | *EpGSTT3* | 648 | 215 | KJ862290 |
| GTT1 | *EpGSTG1* | 705 | 234 | KJ862291 |
|  | *EpGSTG2* | 786 | 261 | KJ862292 |
| EF1Bγ | *EpEF1Bγ1* | 1248 | 415 | KJ862293 |
| Metaxin1-like | *EpMetaxin11* | 1242 | 413 | KJ862294 |
| Zeta | *EpGSTZ1* | 702 | 233 | KJ862295 |
