# Supplementary material for: Identification of Glutathione S-Transferase (GST) Genes from a Dark Septate Endophytic Fungus (Exophiala pisciphila) and Their Expression Patterns under Varied Metals Stress
Source: PLoS One. 2015 Apr 17;10(4):e0123418. doi: 10.1371/journal.pone.0123418 (PMC4401685; doi:10.1371/journal.pone.0123418)
Supplement: S3 Table — (DOC) [file pone.0123418.s004.doc]

**S3 Table. Percentage of identities of amino acid residues within the GSTs of *E. pisciphila***

| GSTclasses | 1 | 2 | 3 | 4 | 5 | 6 | 7 | 8 | 9 | 10 | 11 | 12 | 13 | 14 | 15 | 16 | 17 | 18 | 19 | 20 | 21 | 22 | 23 | 24 |
| --- | --- | --- | --- | --- | --- | --- | --- | --- | --- | --- | --- | --- | --- | --- | --- | --- | --- | --- | --- | --- | --- | --- | --- | --- |
| 1*EpUre2p1* | - | 35.9 | 34.7 | 26.9 | 36.7 | 33.6 | 24.6 | 37.6 | 16.0 | 5.3 | 8.2 | 21.2 | 14.3 | 4.1 | 8.2 | 3.7 | 13.3 | 12.9 | 11.6 | 14.5 | 18.4 | 8.2 | 6.9 | 7.3 |
| 2*EpUre2p2* |  |  | 60.6 | 33.9 | 39.0 | 38.9 | 28.1 | 74.5 | 24.5 | 6.8 | 15.5 | 13.9 | 13.9 | 12.0 | 11.6 | 11.6 | 16.4 | 13.5 | 17.7 | 13.7 | 15.5 | 10.8 | 16.3 | 12.4 |
| 3*EpUre2p3* |  |  |  | 32.4 | 39.1 | 41.8 | 26.3 | 59.3 | 22.6 | 5.9 | 15.1 | 16.2 | 13.0 | 13.8 | 12.6 | 6.3 | 16.0 | 20.8 | 16.3 | 17.1 | 13.8 | 11.1 | 17.0 | 13.3 |
| 4*EpUre2p4* |  |  |  |  | 31.7 | 31.1 | 32.5 | 32.4 | 21.2 | 10.9 | 15.1 | 15.1 | 12.8 | 9.1 | 7.2 | 9.1 | 19.1 | 19.1 | 12.6 | 14.5 | 16.1 | 14.3 | 9.8 | 9.9 |
| 5*EpUre2p5* |  |  |  |  |  | 73.4 | 28.9 | 38.7 | 28.3 | 4.9 | 18.5 | 12.2 | 10.4 | 6.1 | 10.1 | 6.1 | 19.6 | 16.9 | 10.7 | 16.7 | 19.2 | 13.1 | 12.2 | 16.3 |
| 6*EpUre2p6* |  |  |  |  |  |  | 27.6 | 39.8 | 25.9 | 10.7 | 16.8 | 12.7 | 15.2 | 7.4 | 8.6 | 10.7 | 16.4 | 17.4 | 11.2 | 15.8 | 20.9 | 17.6 | 16.0 | 16.3 |
| 7*EpUre2p7* |  |  |  |  |  |  |  | 28.5 | 23.1 | 7.0 | 14.0 | 16.2 | 15.4 | 6.6 | 11.0 | 8.8 | 16.4 | 17.4 | 7.9 | 14.5 | 9.2 | 11.4 | 7.5 | 8.3 |
| 8*EpUre2p8* |  |  |  |  |  |  |  |  | 25.5 | 3.9 | 13.8 | 14.5 | 12.9 | 12.1 | 10.2 | 11.3 | 15.6 | 8.4 | 20.0 | 12.8 | 17.6 | 7.4 | 11.7 | 13.3 |
| 9*EpUre2p9* |  |  |  |  |  |  |  |  |  | 11.8 | 17.5 | 14.6 | 15.1 | 10.8 | 13.7 | 10.8 | 23.6 | 9.6 | 15.1 | 13.7 | 10.4 | 17.5 | 14.6 | 14.2 |
| 10*EpGSTN-31* |  |  |  |  |  |  |  |  |  |  | 12.9 | 6.0 | 6.8 | 10.9 | 3.3 | 8.2 | 2.2 | 4.5 | 13.5 | 9.4 | 11.9 | 8.4 | 7.6 | 10.3 |
| 11*EpGSTN-32* |  |  |  |  |  |  |  |  |  |  |  | 16.4 | 11.6 | 7.3 | 3.4 | 12.5 | 13.3 | 16.3 | 14.9 | 12.9 | 9.9 | 12.9 | 10.3 | 12.9 |
| 12*EpGSTN-33* |  |  |  |  |  |  |  |  |  |  |  |  | 24.3 | 5.1 | 10.0 | 13.0 | 16.0 | 14.6 | 16.3 | 14.5 | 18.4 | 7.0 | 8.9 | 17.6 |
| 13*EpGSTN-34* |  |  |  |  |  |  |  |  |  |  |  |  |  | 13.6 | 9.3 | 11.4 | 11.6 | 16.3 | 9.8 | 7.3 | 16.1 | 5.4 | 13.9 | 21.5 |
| 14*EpGSTN-21* |  |  |  |  |  |  |  |  |  |  |  |  |  |  | 41.2 | 29.7 | 7.6 | 12.9 | 11.2 | 8.5 | 9.2 | 6.3 | 7.0 | 11.6 |
| 15*EpGSTN-22* |  |  |  |  |  |  |  |  |  |  |  |  |  |  |  | 35.8 | 12.0 | 9.0 | 11.2 | 8.5 | 9.6 | 5.1 | 5.9 | 16.3 |
| 16*EpGSTN-23* |  |  |  |  |  |  |  |  |  |  |  |  |  |  |  |  | 9.8 | 15.7 | 6.5 | 10.3 | 16.1 | 3.0 | 1.5 | 5.2 |
| 17*EpGSTT1* |  |  |  |  |  |  |  |  |  |  |  |  |  |  |  |  |  | 25.8 | 12.1 | 12.0 | 14.7 | 11.6 | 11.6 | 8.9 |
| 18*EpGSTT2* |  |  |  |  |  |  |  |  |  |  |  |  |  |  |  |  |  |  | 12.9 | 20.2 | 16.3 | 15.2 | 21.3 | 10.1 |
| 19*EpGSTT3* |  |  |  |  |  |  |  |  |  |  |  |  |  |  |  |  |  |  |  | 6.5 | 14.0 | 13.0 | 13.5 | 18.6 |
| 20*EpGSTG1* |  |  |  |  |  |  |  |  |  |  |  |  |  |  |  |  |  |  |  |  | 25.2 | 14.5 | 13.7 | 10.3 |
| 21*EpGSTG2* |  |  |  |  |  |  |  |  |  |  |  |  |  |  |  |  |  |  |  |  |  | 8.8 | 11.1 | 4.7 |
| 22*EpEF1Bγ1* |  |  |  |  |  |  |  |  |  |  |  |  |  |  |  |  |  |  |  |  |  |  | 10.2 | 7.7 |
| 23*EpMetaxin11* |  |  |  |  |  |  |  |  |  |  |  |  |  |  |  |  |  |  |  |  |  |  |  | 17.2 |
| 24*EpGSTZ1* |  |  |  |  |  |  |  |  |  |  |  |  |  |  |  |  |  |  |  |  |  |  |  | - |
