# Supplementary material for: Identification of Glutathione S-Transferase (GST) Genes from a Dark Septate Endophytic Fungus (Exophiala pisciphila) and Their Expression Patterns under Varied Metals Stress
Source: PLoS One. 2015 Apr 17;10(4):e0123418. doi: 10.1371/journal.pone.0123418 (PMC4401685; doi:10.1371/journal.pone.0123418)
Supplement: S4 Table — (DOC) [file pone.0123418.s005.doc]

**S4 Table. Average values of relative expression of the 24 *EpGST*s**

| *EpGST*s | Average relative expression | | | |
| --- | --- | --- | --- | --- |
|  | Cd stress | Pb stress | Cu stress | Zn stress |
| *EpUre2p1* | 0.713813 | 2.93263 | 3.841148 | 3.226339 |
| *EpUre2p2* | 0.617165 | 11.86836 | 0.607978 | 37.68762 |
| *EpUre2p3* | 3.780785 | 9.27343 | 2.424911 | 0.861051 |
| *EpUre2p4* | 1.002846 | 2.701209 | 0.968435 | 0.548485 |
| *EpUre2p5* | 2.052713 | 4.559105 | 9.967014 | 3.565301 |
| *EpUre2p6* | 0.921052 | 2.914725 | 0.233963 | 0.509552 |
| *EpUre2p7* | 0.281309 | 3.596031 | 1.169646 | 0.592946 |
| *EpUre2p8* | 2.172588 | 2.340243 | 23.63245 | 4.79482 |
| *EpUre2p9* | 2.310747 | 1.373958 | 3.108642 | 0.695838 |
| *EpGSTN-31* | 4.819948 | 2.064281 | 5.654171 | 1.120709 |
| *EpGSTN-32* | 2.711861 | 9.749444 | 13.60988 | 2.688201 |
| *EpGSTN-33* | 1.856536 | 4.806166 | 3.520522 | 1.081185 |
| *EpGSTN-34* | 1.542586 | 2.548747 | 97.33507 | 0.677173 |
| *EpGSTN-21* | 3.284524 | 13.0958 | 15.67851 | 10.70771 |
| *EpGSTN-22* | 2.208019 | 2.863478 | 27.97825 | 4.290325 |
| *EpGSTN-23* | 2.002482 | 5.638664 | 16.74891 | 4.324026 |
| *EpGSTT1* | 0.09138 | 7.704449 | 1.30049 | 1.86726 |
| *EpGSTT2* | 1.004716 | 3.100446 | 7.499737 | 2.449004 |
| *EpGSTT3* | 1.575205 | 9.395125 | 36.77413 | 10.94535 |
| *EpGSTG1* | 2.278874 | 12.03658 | 6.269635 | 2.601654 |
| *EpGSTG2* | 2.378793 | 1.837568 | 13.25287 | 1.655539 |
| *EpEF1Bγ1* | 1.075639 | 2.468676 | 3.001773 | 2.285198 |
| *EpMetaxin11* | 2.630828 | 25.91198 | 18.36037 | 3.367533 |
| *EpGSTZ1* | 4.058648 | 4.881843 | 12.04333 | 7.467339 |
