# Supplementary material for: Identification of Glutathione S-Transferase (GST) Genes from a Dark Septate Endophytic Fungus (Exophiala pisciphila) and Their Expression Patterns under Varied Metals Stress
Source: PLoS One. 2015 Apr 17;10(4):e0123418. doi: 10.1371/journal.pone.0123418 (PMC4401685; doi:10.1371/journal.pone.0123418)
Supplement: S5 Table — (DOC) [file pone.0123418.s006.doc]

**S5 Table. Percentage of identities of amino acid residues within the 17 members in N-3 class**

| GST | 1 | 2 | 3 | 4 | 5 | 6 | 7 | 8 | 9 | 10 | 11 | 12 | 13 | 14 | 15 | 16 | 17 |
| --- | --- | --- | --- | --- | --- | --- | --- | --- | --- | --- | --- | --- | --- | --- | --- | --- | --- |
| 1*EpGSTN-32* | - | 50.0 | 53.9 | 52.2 | 16.4 | 15.1 | 15.1 | 11.6 | 20.7 | 15.1 | 15.9 | 17.7 | 9.9 | 8.6 | 12.9 | 6.0 | 7.3 |
| 2*Parvibaculum lavamentivorans* |  |  | 52.3 | 56.7 | 14.6 | 16.1 | 16.9 | 18.4 | 21.5 | 18.4 | 19.9 | 17.6 | 6.1 | 11.5 | 17.2 | 5.7 | 13.0 |
| 3*Methylobacterium populi* |  |  |  | 49.6 | 17.7 | 17.7 | 15.8 | 12.7 | 15.0 | 20.0 | 20.8 | 17.3 | 6.9 | 15.8 | 18.1 | 12.3 | 15.0 |
| 4*Chlorogloeopsis* sp |  |  |  |  | 13.7 | 17.2 | 17.6 | 17.9 | 20.6 | 17.2 | 22.1 | 19.5 | 5.7 | 8.8 | 19.1 | 8.0 | 9.9 |
| 5*EpGSTN-33* |  |  |  |  |  | 40.1 | 39.4 | 24.3 | 26.2 | 28.2 | 31.4 | 30.0 | 7.6 | 6.0 | 10.9 | 8.5 | 4.8 |
| 6*Arthroderma gypseum* |  |  |  |  |  |  | 56.6 | 36.1 | 34.4 | 34.3 | 37.9 | 34.3 | 8.2 | 6.8 | 8.0 | 6.9 | 5.4 |
| 7*Paracoccidioides brasiliensis* |  |  |  |  |  |  |  | 32.9 | 34.7 | 37.1 | 41.4 | 35.7 | 8.2 | 8.2 | 5.6 | 11.3 | 7.8 |
| 8*EpGSTN-34* |  |  |  |  |  |  |  |  | 74.3 | 60.7 | 60.0 | 56.0 | 1.8 | 6.8 | 8.9 | 7.1 | 9.3 |
| 9*Exophiala dermatitidi*s |  |  |  |  |  |  |  |  |  | 61.1 | 63.2 | 55.6 | 3.2 | 9.1 | 5.9 | 9.1 | 6.0 |
| 10*Colletotrichum higginsianum* |  |  |  |  |  |  |  |  |  |  | 68.6 | 52.3 | 1.8 | 8.9 | 8.6 | 6.4 | 3.2 |
| 11*Nectria haematococca* |  |  |  |  |  |  |  |  |  |  |  | 53.1 | 6.8 | 12.9 | 8.6 | 8.6 | 8.2 |
| 12*Exophiala dermatitidis* |  |  |  |  |  |  |  |  |  |  |  |  | 8.3 | 10.8 | 15.5 | 10.5 | 13.4 |
| 13*Exophiala dermatitidis* |  |  |  |  |  |  |  |  |  |  |  |  |  | 50.1 | 46.3 | 44.6 | 21.0 |
| 14*EpGSTN-31* |  |  |  |  |  |  |  |  |  |  |  |  |  |  | 54.8 | 52.9 | 26.1 |
| 15*Coniosporium apollinis* |  |  |  |  |  |  |  |  |  |  |  |  |  |  |  | 57.0 | 25.8 |
| 16*Zymoseptoria tritici* |  |  |  |  |  |  |  |  |  |  |  |  |  |  |  |  | 28.5 |
| 17E*xophiala dermatitidis* |  |  |  |  |  |  |  |  |  |  |  |  |  |  |  |  | - |
