# Supplementary material for: Identification of Glutathione S-Transferase (GST) Genes from a Dark Septate Endophytic Fungus (Exophiala pisciphila) and Their Expression Patterns under Varied Metals Stress
Source: PLoS One. 2015 Apr 17;10(4):e0123418. doi: 10.1371/journal.pone.0123418 (PMC4401685; doi:10.1371/journal.pone.0123418)
Supplement: S6 Table — (DOC) [file pone.0123418.s007.doc]

***S6 Table. Percentage of identities of amino acid residues within the 13 GSTs in*** Exophiala dermatitidis

|  | 1 | 2 | 3 | 4 | 5 | 6 | 7 | 8 | 9 | 10 | 11 | 12 | 13 |
| --- | --- | --- | --- | --- | --- | --- | --- | --- | --- | --- | --- | --- | --- |
| 1EHY57213.1 | - | 6.3 | 8.4 | 8.8 | 6.6 | 4.1 | 5.5 | 7.2 | 7.6 | 4.1 | 10.6 | 21.0 | 7.3 |
| 2EHY52537.1 |  |  | 7.7 | 4.3 | 5.3 | 10.0 | 7.3 | 13.5 | 3.6 | 9.0 | 6.0 | 9.9 | 11.0 |
| 3EHY59473.1 |  |  |  | 37.5 | 42.1 | 10.3 | 7.3 | 6.3 | 10.5 | 4.9 | 8.1 | 12.9 | 7.3 |
| 4EHY51929.1 |  |  |  |  | 38.9 | 9.7 | 10.9 | 11.0 | 17.0 | 2.6 | 7.7 | 6.6 | 6.1 |
| 5EHY52776.1 |  |  |  |  |  | 13.2 | 12.4 | 10.1 | 15.2 | 5.8 | 7.7 | 4.2 | 6.9 |
| 6EHY60173.1 |  |  |  |  |  |  | 16.8 | 12.7 | 55.6 | 8.2 | 25.5 | 6.0 | 15.9 |
| 7EHY60945.1 |  |  |  |  |  |  |  | 14.8 | 17.9 | 14.6 | 12.8 | 7.3 | 36.2 |
| 8EHY59024.1 |  |  |  |  |  |  |  |  | 13.9 | 13.9 | 12.3 | 13.5 | 13.5 |
| 9EHY54854.1 |  |  |  |  |  |  |  |  |  | 13.4 | 20.9 | 13.4 | 18.7 |
| 10EHY56652.1 |  |  |  |  |  |  |  |  |  |  | 10.6 | 3.3 | 10.6 |
| 11EHY55296.1 |  |  |  |  |  |  |  |  |  |  |  | 10.6 | 7.2 |
| 12EHY56712.1 |  |  |  |  |  |  |  |  |  |  |  | 6.1 |  |
| 13EHY56510.1 |  |  |  |  |  |  |  |  |  |  |  |  | - |
